# From Processing to Pathways: Advancing Cardiovascular Nutrition Equity through Mechanism-Based Front-of-Package Labeling

**DOI:** 10.1016/j.jacadv.2025.102515

**Published:** 2026-03-17

**Authors:** Kim Allan Williams

**Affiliations:** Department of Internal Medicine Ambulatory Care Building, University of Louisville School of Medicine, Louisville, Kentucky, USA

**Keywords:** cardiovascular nutrition, food environment, front-of-package labeling, preventive health, ultra-processed foods

**Figure:**
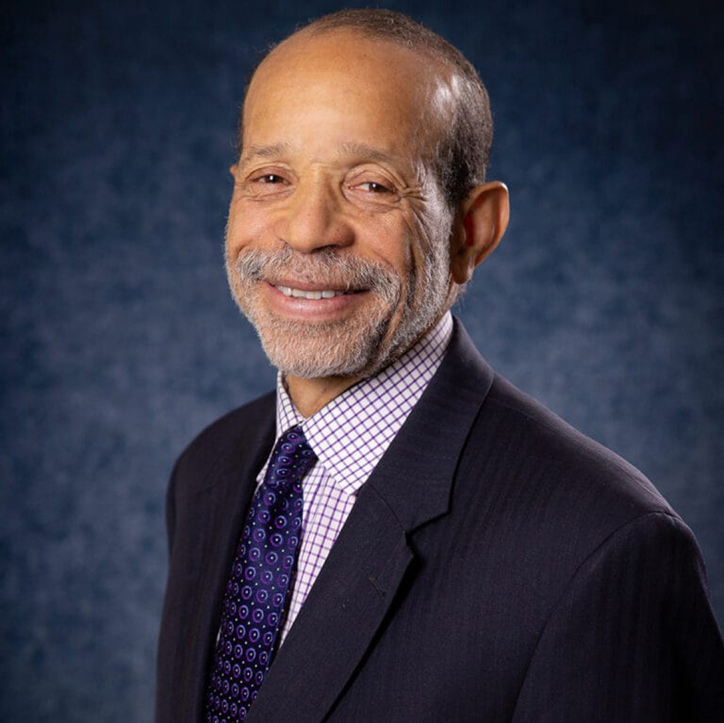

The study by Haidar et al^1^ in this issue of *JACC* adds an important dimension to the rapidly growing literature linking ultraprocessed food (UPF) consumption with adverse cardiovascular outcomes. Using the richly characterized MESA (Multiethnic Study of Atherosclerosis), the authors confirm a graded association between higher UPF consumption and incident atherosclerotic cardiovascular disease (ASCVD) and identify a statistically significant interaction suggesting that Black Americans may experience greater ASCVD risk per unit of UPF exposure than non-Black participants. Their analysis is careful, methodologically transparent, and responsive to recent calls from the 2025 Dietary Guidelines Advisory Committee for diverse U.S.-based evidence. The work stands as one of the strongest examinations to date of UPFs in a heterogeneous American cohort.^1^

Although the findings are compelling, this manuscript also highlights deeper conceptual challenges in how our field defines and operationalizes UPFs—and, importantly, how those limitations intersect with discussions of race, diet, and cardiovascular health.

## Is “processing” the exposure: or is processing a proxy?

The study uses the NOVA classification system, now the most widely recognized framework for categorizing foods by the extent of industrial processing.^2^ NOVA does not classify foods based on nutrient quality or healthfulness; it categorizes foods based solely on how they are manufactured. This distinction is critical because it leads to well-recognized inconsistencies.

For example, NOVA categorizes unsweetened soy milk—a product shown to improve blood pressure, lipids, endothelial function, and insulin sensitivity—as a UPF,^3^, ^4^, ^5^ whereas eggs, red meat, full-fat dairy, and processed meats are classified as “unprocessed or minimally processed,” despite robust evidence linking each to increased cardiovascular morbidity and mortality.^6^, ^7^, ^8^ These inconsistencies illustrate that “processing” is an imperfect surrogate for cardiometabolic harm.

In the present analysis, the UPFs most strongly associated with ASCVD were sugary foods, whereas associations for meats, breads, savory snacks, mixed dishes, and sugar-sweetened beverages attenuated or disappeared with full adjustment.^1^ This heterogeneity reflects the inherent limitations of NOVA: it aggregates disparate foods into a single exposure category without distinguishing nutrient profiles, additives, or mechanisms of harm.

Thus, clinicians should avoid attributing causal meaning to “processing” alone when nutrient composition, sodium load, cholesterol, saturated fat, sugar content, food-matrix disruption, and environmental context remain more plausible drivers.

## Does NOVA add value: or NOVAlue?

### Why classification without nutrient context misleads cardiovascular science

This study underscores a central problem: NOVA offers a classification, but not a mechanism. It sorts foods by industrial process, not by the biological pathways that shape ASCVD risk. As a result, NOVA often obscures meaningful differences between harmful and health-supportive foods.

A more effective approach—one endorsed by the American College of Cardiology^9^ and upcoming federal initiatives—prioritizes nutrient exposures known to drive cardiovascular disease: added sugars, cholesterol, sodium, saturated fat, and highly refined starches, not simply “processing.”

## Why do UPFs appear more harmful in Black Americans? A more complete explanation

The UPF × race interaction is the most consequential finding in Haidar et al.^1^ Although “processing” is not inherently more biologically toxic to Black individuals, population-level physiologic differences relevant to dietary exposures merit attention. Black adults, on average, exhibit greater sodium sensitivity and a higher prevalence of low-renin, volume-expanded hypertension, phenotypes consistently documented in physiologic trials.^10^ These features amplify the hemodynamic consequences of high-sodium UPF subtypes—fried foods, refined-flour entrées, processed meats—that are disproportionately available and aggressively marketed in predominantly Black communities.^11^

### Measurement limitations further influence this interaction

Food-frequency questionnaires (FFQs) capture frequency but render only semiquantitative estimates of portion size. If, for example, Black participants consumed much larger portions of sodium- or sugar-dense UPFs, FFQs would systematically underestimate exposure. In addition, although the authors adjusted for body mass index, hypertension, and diabetes, these variables were treated only as binary indicators rather than continuous variables, and would not capture disease severity, visceral adiposity, degree of glycemia, insulin resistance, or subclinical organ damage.

### Equally important is what the models did *not* adjust for

The analysis omitted socioeconomic status, neighborhood deprivation, food insecurity, chronic stress, and validated measures of *allostatic load*—factors that differ substantially by race within MESA and strongly influence both dietary exposure and physiologic vulnerability. Socioeconomic status and chronic stress shape sodium handling, endothelial function, sympathetic tone, visceral adiposity, and inflammatory pathways.^12^^,^^13^ Without accounting for these structural determinants, the stronger UPF–ASCVD association among Black adults likely reflects differential structural environments, not differential biological susceptibility.

In this sense, UPFs function not as primary etiologic agents but as amplifiers of structural inequity.

## The challenge of measurement: FFQs and misclassification

Haidar et al^1^ acknowledge that the MESA FFQ was not designed to classify foods by processing level. Retrospective application of NOVA introduces misclassification—a limitation widely recognized in UPF literature.^14^^,^^15^ Foods such as “hamburger,” “cold cereal,” or “fried fish” can vary substantially in preparation, additives, and nutrient profile—details not captured by FFQs.

This highlights the need for modern, mechanistic dietary assessment tools, including barcode-linked purchase data, biomarkers of additives or matrix disruption, and computational approaches to capturing portion-size variability.

## UPFs, health equity, and the future of National Food Labeling Policy

The stronger UPF-ASCVD associations among Black Americans also highlight the urgency of structural solutions, including transparent front-of-package (FOP) labeling. The American College of Cardiology’s recent Expert Consensus Decision Pathway^9^ supporting Food and Drug Administration-led FOP labeling provides a pragmatic, equity-oriented framework that communicates the nutrients most relevant to ASCVD—sodium, added sugars, cholesterol and saturated fat—rather than the degree of processing per se.^1^ Unlike NOVA, which can classify both harmful and health-supportive foods as “ultraprocessed,” FOP labels furnish consumers with concise, biologically meaningful information at the point of purchase. For communities disproportionately exposed to targeted marketing, constrained food environments, and chronic stress, FOP labeling represents a structural tool that empowers healthier choices and counters the very inequities amplified in the present study. The path to equity lies not in labeling foods as “processed,” but in labeling them with the information that matters.

This is where national nutrition policy is headed—and should be headed.

## Where do we go from here?

*First, UPF research must evolve beyond NOVA.*

Mechanistic risk arises from excess sodium, added sugars, saturated fat, cholesterol, refined starch, nitrites, and food-matrix disruption—not from “processing” in the abstract.

*Second, interpretation of differential UPF risk must embed structural inequity.*

The interaction observed in MESA likely reflects environmental constraints, targeted marketing, neighborhood food environments, chronic stress, and sodium-sensitive physiology—not inherent vulnerability.

*Third, clinical translation demands nuance.*

The solution is not to treat all UPFs as unhealthy; rather, clinicians should differentiate between health-supportive and harmful UPFs and emphasize known cardiometabolic risks such as added sugars and sodium.

## Conclusions

Haidar et al^1^ provide an important contribution to cardiovascular nutrition science, demonstrating that UPF consumption is associated with ASCVD and that structural, physiologic, and measurement-related factors amplify this association for Black Americans. At the same time, the study illustrates the limits of NOVA as an exposure metric and highlights the need for labeling systems—such as Food and Drug Administration’s proposed FOP labels—that communicate what matters biologically and equitably.

The future of cardiovascular nutrition lies not in classifying foods by how they are processed, but in labeling foods by the pathways through which they influence risk.

## Funding support and author disclosures

The authors have reported that they have no relationships relevant to the contents of this paper to disclose.
